# Long term follow-up of *Trypanosoma cruzi* infection and Chagas disease manifestations in mice treated with benznidazole or posaconazole

**DOI:** 10.1371/journal.pntd.0008726

**Published:** 2020-09-24

**Authors:** Claudia Magalhães Calvet, Tatiana Araújo Silva, Diane Thomas, Brian Suzuki, Ken Hirata, Jair Lage Siqueira-Neto, James H. McKerrow

**Affiliations:** 1 Center for Discovery and Innovation in Parasitic Diseases, Skaggs School of Pharmacy and Pharmaceutical Sciences, University of California, San Diego, California, United States of America; 2 Cellular Ultrastructure Laboratory, Oswaldo Cruz Institute (IOC), FIOCRUZ, Rio de Janeiro, RJ, Brazil; Yale University School of Medicine, UNITED STATES

## Abstract

Chagas' Disease, caused by the protozoan parasite *Trypanosoma cruzi*, is responsible for up to 41% of the heart failures in endemic areas in South America and is an emerging infection in regions of North America, Europe, and Asia. Treatment is suboptimal due to two factors. First, the lack of an adequate biomarker to predict disease severity and response to therapy; and second, up to 120-days treatment course coupled with a significant incidence of adverse effects from the drug currently used. Because the disease can manifest itself clinically a few years to decades after infection, controversy remains concerning the suitability of current drug treatment (benznidazole), and the efficacy of alternative drugs (e.g. posaconazole). We therefore followed the clinical course, and PCR detection of parasite burden, in a mouse model of infection for a full year following treatment with benznidazole or posaconazole. Efficacy of the two drugs depended on whether the treatment was performed during the acute model or the chronic model of infection. Posaconazole was clearly superior in treatment of acute disease whereas only benznidazole had efficacy in the chronic model. These results have important implications for the design and analysis of human clinical trials, and the use of specific drugs in specific clinical settings.

## Author summary

Chagas disease is a parasitic infection that can be incapacitating, leading to heart failure with risk of sudden death. Currently, only one drug treatment is available, Benznidazole, but it demands a long period of treatment, has variable efficacy and leads to serious side effects that can lead to discontinuation of the treatment. An alternative therapy, the anti-fungal drug, Posaconazole, was repurposed for treatment of Chagas disease, but its use has been controversial with conflicting results in studies from different groups. In our approach, we followed the parasitic infection, disease symptoms and persistence of the pathogen in mice for a full year after treatment with Benznidazole or Posaconazole. Posaconazole treatment was more effective in the early infection (acute disease) whereas only Benznidazole had efficacy in the chronic model. This information can have important implications for the design and analysis of human clinical trials, and the use of specific drugs in specific clinical settings.

## Introduction

Chagas disease, caused by the parasite *Trypanosoma cruzi*, is responsible for up to 41% of the heart failures in endemic areas in South America[1,2] and an emerging infection in several northern hemisphere countries [3,4]. The annual financial burden of Chagas disease is estimated to be US $7.2 billion, more than the cost of the majority of cancers[2,5]. The most commonly used drug in South America is benznidazole, a nitro-containing compound that generates free radicals lethal to the parasite. Benznidazole is only approved for pediatric use (<12 years) in the United States. Current dosing regimens are for at least 60 days and there are frequent adverse effects including rash, epigastric pain, pruritus, nausea and abdominal swelling [6]. In the search for alternative therapy, the anti-fungal drug, Posaconazole, was repurposed for treatment of *T*. *cruzi* infections[7,8]. Posaconazole is clearly efficacious *in vitro*[9] and in mouse models of infection[10,11]. While there was a reported cure of acute Chagas disease with posaconazole treatment following disease reactivation due to immunossupression[12], clinical trials of posaconazole for the treatment of *T*. *cruzi* infected patients were reported as “failures” [13]. This latter conclusion was based on the failure of posaconazole to achieve sterile cure as defined by negative PCR for the parasite at 3–10 months post treatment ^6,^[14]. The concern was that infection had been reactivated and this will ultimately lead to cardiac infection and cardiac failure.

The BENEFIT trial, a prospective study of treatment of patients with chronic, “determinate” Chagas Disease reported that benznidazole failed to halt disease progression [15]. Controversy remains as to whether the lack of benznidazole efficacy reflects parasite strain differences and/or the fact that the patients already had manifestations of cardiac failure. It has been argued that different strains of *T*. *cruzi*, existing in different geographical areas, may respond differently to specific drug therapy[16]. A non-randomized study of benznidazole treatment showed efficacy as delineated by lack of disease progression in patients with no cardiac failure[17]. Therefore, it is still unclear whether benznidazole treatment earlier in disease progression (chronic indeterminate patients) might slow or halt progression to cardiac failure. Finally, Lewis et al[18] reported the presence of residual, “cryptic” parasites in the gastrointestinal track of infected mice, and recent reports suggested that *T*. *cruzi* might enter a “dormant” or non/low replicating form during its intracellular cycle[19]. Therefore, it was speculated that specific parasite populations in the host might be tolerant to drugs requiring active parasite replication.

In the absence of a validated biomarker for predicting disease progression or response to therapy, PCR has been used to monitor the presence or absence of *T*.*cruzi* infection. However, it has been argued that sterile cure (PCR negative) may not be required for halting the progression to cardiac failure in Chagas disease [20]. To date, no prospective study has evaluated whether the actual clinical course of Chagas disease is altered by significantly reducing parasite load without achieving sterile cure. The lack of such data is in large part attributable to the length of time required for a patient in the chronic, indeterminate stage of Chagas disease to progress to overt cardiac failure.

To evaluate the efficacy of drug therapy to suppress disease manifestations, we performed treatment in acute and chronic models of *T*. *cruzi* infection. Benznidazole or posaconazole were administered to mice after acute infection with *T*. *cruzi* Sylvio X10/7, or in the early chronic phase, 60 days after infection with *T*. *cruzi* Brazil. We then followed for one year survival, weight, activity, feeding, and gross appearance of the mice, also analyzing parasite load and histopathology in the heart and GI tract. In the acute model, posaconazole treatment outcomes were superior to benznidazole, while in the chronic phase, benznidazole treatment was more beneficial.

## Material and methods

### Ethics statement

All procedures involving animals were approved by the Institutional Animal Care and Use Committee from UCSD, protocol number 14187 to Jair L Siqueira-Neto. The vivarium where mice were housed during experiments is fully accredited by the Association for Assessment and Accreditation of Laboratory Animal Care International. The studies complied to the Animal Welfare Act and Regulations (USDA/APHIS), Public Health Service Policy on Humane Care and Use of Laboratory Animals (OLAW/PHS Policy, AVMA Guidelines for the Euthanasia of Animals: 2013 Edition, and the guidelines in the Guide for the Care and Use of Laboratory Animals, National Research Council, 2011.

### Animals

Male C57/Bl6 mice, 6 weeks old, were purchased from Charles River Laboratories. Mice were housed at a maximum number of 5 animals per cage and kept in a conventional room at 20 to 24°C under a 12 h/12 h light/dark cycle. The animals were provided with sterilized water and chow ad libitum.

### Parasite strains

*T*. *cruzi* Sylvio X10/7 was used to evaluate the acute stage of infection and Brazil strain the chronic stage. Sylvio X/10-7 infection leads to acute cardiomyopathy, indicated by abdominal ascites and inflammatory infiltrates in the heart, resulting in acute death[21]. Brazil strain was chosen to study the chronic phase since several reports show that mice survive acute infection, and develop pathophysiological alterations in the heart during the chronic stage of infection, showing chronic inflammation and hypertrophy [22,23].

### Parasite culture and infection of mice

*T*. *cruzi* Sylvio X10/7 and Brazil strain were maintained in C2C12 myoblast culture. After 5–7 days, trypomastigotes released in the supernatant were collected by centrifugation for 15 min at 3300 rpm, re-suspended in DMEM, and used to infect mice by intraperitoneal injection with 10^6^ (Sylvio X10/7) or 1x10^4^ (Brazil) trypomastigote forms of *T*. *cruzi*/ mice. The acute infection with Sylvio X10/7 was performed in two independent experiments.

### Treatment protocols

Benznidazole (Sigma, St Louis, MO) and Posaconazole (purified from oral suspension Noxafil) were solubilized in 10% solutol (Kolliphor, Sigma, St Louis, MO), and administered once a day orally, at 100 mg/kg for benznidazole, and 20 mg/kg for posaconazole. A solution of 10% solutol (just the vehicle) was administered to control, *T*. *cruzi*- infected groups. The treatment of mice acutely infected with *T*. *cruzi* Sylvio X10/7 strain started 6 days post-infection (dpi). For analysis at the chronic stage of infection, mice infected with *T*. *cruzi* Brazil strain were treated starting at 60 days post infection. Mice were treated for 21 days. In both approaches, survival, general health, and the weight of mice was monitored for one year after the end of treatment.

### *T*. *cruzi* detection by quantitative PCR

One year after completion of treatment, mice were euthanized and heart and sections of GI tract (isolated from duodenum, cecum and colon) were removed, cleaned and briefly washed in PBS. The GI tract samples were pooled and snap frozen in liquid nitrogen. 50 mg of tissue was homogenized by bead agitation using a ZR BashingBead Lysis Tube (2.0 mm, Zymo Research, Irvine, CA) and DNA was purified using Quick-DNA Miniprep Plus Kit (Zymo Research). Quantitative PCR was performed as described elsewhere [24], adapting protocols from other authors [25,26]. Briefly, 180 ng of DNA was used as template for qPCR using Lightcycler 480 Sybr green I Master mix (Roche) on a Stratagene Mx3005P RT-PCR thermocycler. Parasite satellite DNA region was detected with primers ASTCGGCTGATCGTTTTCGA and AATTCCTCCAAGCAGCGGATA and mouse TNFα with primers TCCCTCTCATCAGTTCTATGGCCCA and CAGCAAGCATCTATGCACTTAGACCCC. Thermal profile consisted of 95°C for 10 min; 40 cycles at 95°C for 30s; 58°C for 60 s and 72°C for 60s. To determine parasite burden in 50 mg of tissue, a standard curve was established with uninfected mice heart samples spiked with 2x10^7^ *T*. *cruzi* epimastigotes. The standard curve was generated through serial 10-fold dilutions in DNA from uninfected mice, resulting in a curve ranging from 2 to 200,000 parasite equivalents. *T*. *cruzi* satellite DNA values were normalized with mouse TNF-α detection, and the delta Ct from these two genes was calculated for all samples. The standard curve was performed at each measurement, and the parasite load for each sample was calculated from the equation of the linear regression of the curve. Mice were considered positive for *T*. *cruzi* infection if the parasite burden was higher than average plus 3 standard deviations versus uninfected mice. Data were analyzed using Microsoft Excel and GraphPad Prism 6.

### Histology

Upon euthanasia, the heart was cut in half in sagittal orientation, placed in cryomolds, embedded in Tissue-Tek (O.C.T., Sakura Finetek) and snap frozen in liquid nitrogen. Samples were sectioned in a cryostat, fixed in buffered formalin and stained with hematoxylin and eosin. The slides were scanned using a Nanozoomer Slide Scanner (Hamamatsu Photonics, NJ, USA) and images were obtained through NDP viewer software (Hamamatsu Photonics, NJ, USA).

### Histopathology analysis

Levels of inflammation were quantified as previously described [27]. Briefly, 5 random images of mouse heart (10x magnification) were obtained from each animal, comprising most of heart section area. Lymphocyte nuclei was segmented through the Particle Analyzer Image processing plugin from FIJI software [28], and lymphocyte nuclei were counted.

### Echocardiography in chronic disease model

For evaluation of cardiac condition and performance, echocardiographic analysis was performed using a micro-imaging ultrasound system, the Vevo 2100, operating at 30–40 MHz (available at the Seaweed Canyon Cardiovascular Physiology Laboratory, Institute for Molecular Medicine, UCSD). Before the procedure, the fur from the thoracic region of mice was removed and the animals were subjected to light anesthesia with isoflurane (1.25–1.5% in 100% oxygen). Small electrodes for electrocardiogram recordings were inserted in upper and lower limbs, and the echocardiographic probe gently placed on the chest surface. Doppler tracing for trans-aortic and mitral valve flows were also recorded. Two-dimensional images were obtained at a minimum depth setting of 2cm with zoom enhanced imaging function activated and a region of interest adjusted to the heart size. The M-mode and Doppler tracings were recorded at a sweep speed of 150mm/sec with analyses performed off-line. The bi-dimensional images were reconstructed after a cardiac cycle allowing visualization of myocardial function, and measurement of size and wall stress.

### Statistical analysis

Mann-Whitney non-parametric test was used for comparison of experimental data from quantitative PCR measurement of parasite load in mice. Values were considered statistically significant when p≤ 0.05.

## Results

### Acute Chagas disease model

**Fig 1:**
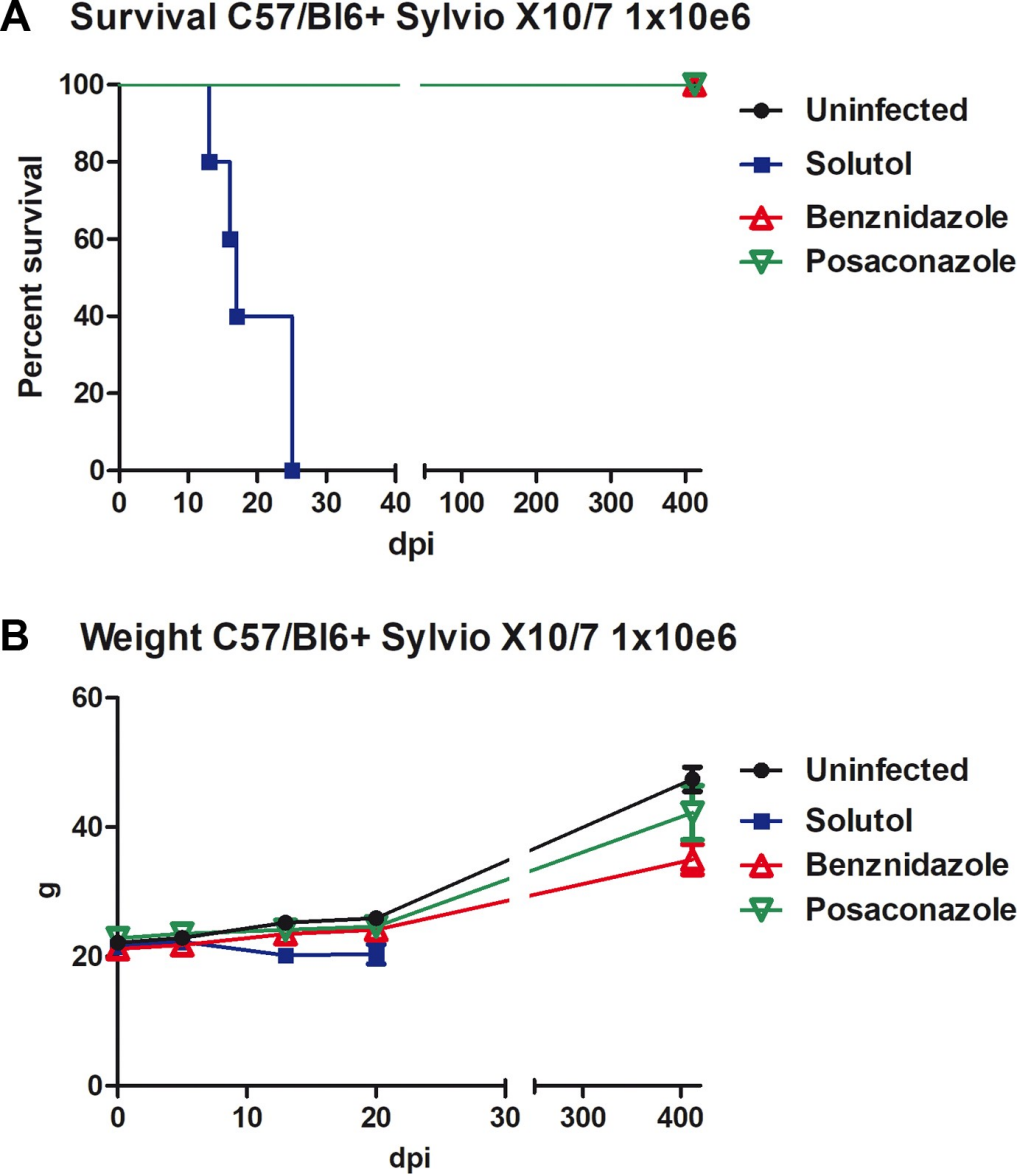
(A) Survival of mice infected with Silvio X10/7 strain of *T*.*cruzi* in a model of acute Chagas Disease. Note all vehicle alone (solutol) treated mice died by 25 days, whereas both benznidazole and 9/10 posaconazole-treated mice survived one year. One posaconazole-treated mouse died early likely from gavage trauma. (B) Posaconazole-treated mice were statistically similar to uninfected mice in weight gain over one year. While benznidazole-treated mice also gained weight, they did so at a significantly slower pace.

Following infection with the *T*. *cruzi*, Sylvio X10/7 strain, all untreated and vehicle treated mice died after 25 days. In this model of infection, untreated mice also developed abdominal ascites, which is a sign of acute cardiomyopathy. All benznidazole and 9 out of 10 posaconazole-treated mice survived for one year (Fig 1A). One posaconazole-treated mouse died early (21 days), consistent with trauma from gavage. All treated mice were active, ate appropriately, and showed no signs of ascites and no gross alterations of appearance such as ruffled hair, skin lesions, or disturbances of gait. Infected and untreated mice lost weight before dying of acute infection (Fig 1B). All surviving mice gained weight steadily over the monitored period. Mice in the benznidazole-treated group gained less weight and therefore weighed significantly less than controls or posaconazol- treated mice at one year (Fig 1B).

**Fig 2:**
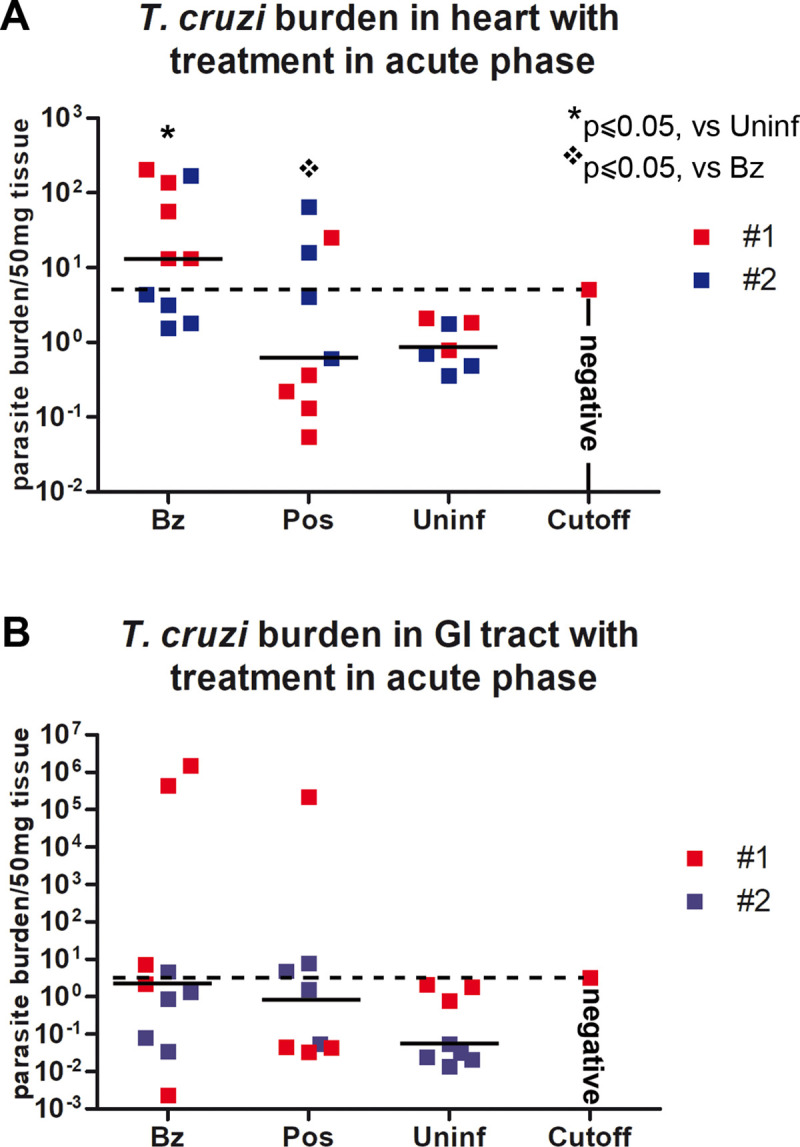
Parasite burden in heart (A) and G.I. tract (B) in acute model of infection as assessed by PCR (see Methods). Six of nine posaconazole-treated mice had negative PCR as did the uninfected controls. One of the PCR-positive mice treated with posaconazole had no detectable cardiac inflammation (Fig 3). Combined results from two independent studies color coded as #1 (red) and #2 (blue).* Statistically significant versus Uninfected samples, p≤ 0.05; ❖ Statistically significant versus benznidazole treated mice, p≤ 0.05.

Analysis of parasite load in heart and GI tract by qPCR revealed that 6/10 benznidazole-treated mice still harbored Sylvio X10/7 parasites in cardiac tissue after one year post infection (Fig 2A). 6/9 posaconazole-treated mice were PCR negative in cardiac tissue as were all 7 uninfected mice. 4/10 benznidazole-treated and 3/9 posaconazole-treated mice were PCR positive in the gastrointestinal tract tissue one year post-infection (Fig 2B). The same posaconazole-treated mice with positive PCR in cardiac tissue had positive PCR in gastrointestinal samples.

**Fig 3:**
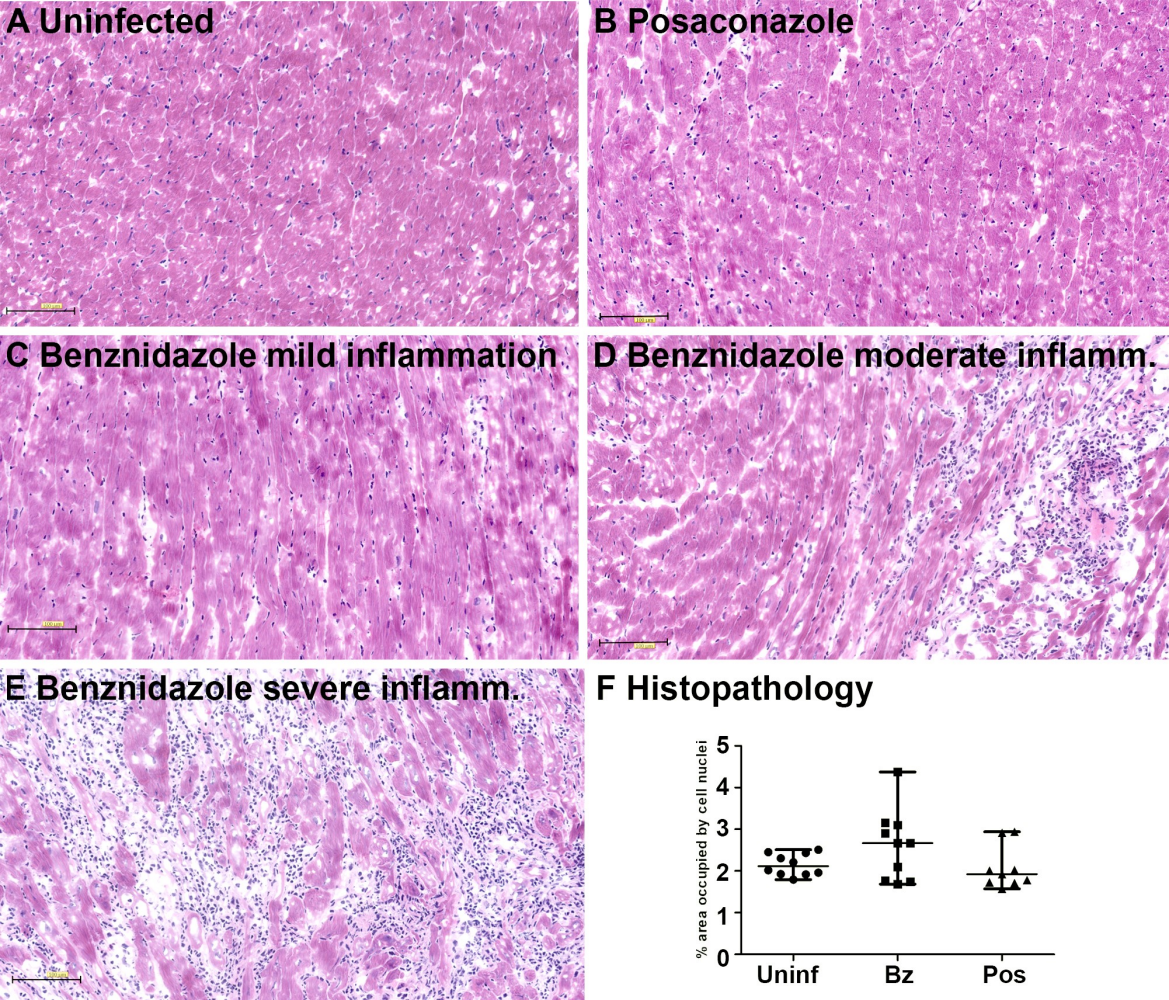
(A-E) Inflammatory infiltrates associated with parasite infection, in heart muscle. Heart muscle fibers are pink with eosin-stain while the inflammatory infiltrate associated with nests of parasites is recognized by the presence of dark-blue lymphocytes. (F) Quantification of inflammatory infiltrate by number of lymphocytes through image processing (see Methods).

Histopathologic analysis of cardiac tissue identified significant inflammatory reactions (predominantly lymphocytes) in 6/10 benznidazole treated but only in 1/9 of the posaconazole treated mice at one year post infection (Fig 3). Histopathologic analysis of gastrointestinal tissue revealed no significant alteration after infection or after treatment. Particularly noteworthy was one posaconazole-treated mouse with positive PCR but no detectable inflammation by histopathology of heart and GI tract.

### Chronic Chagas disease model

**Fig 4:**
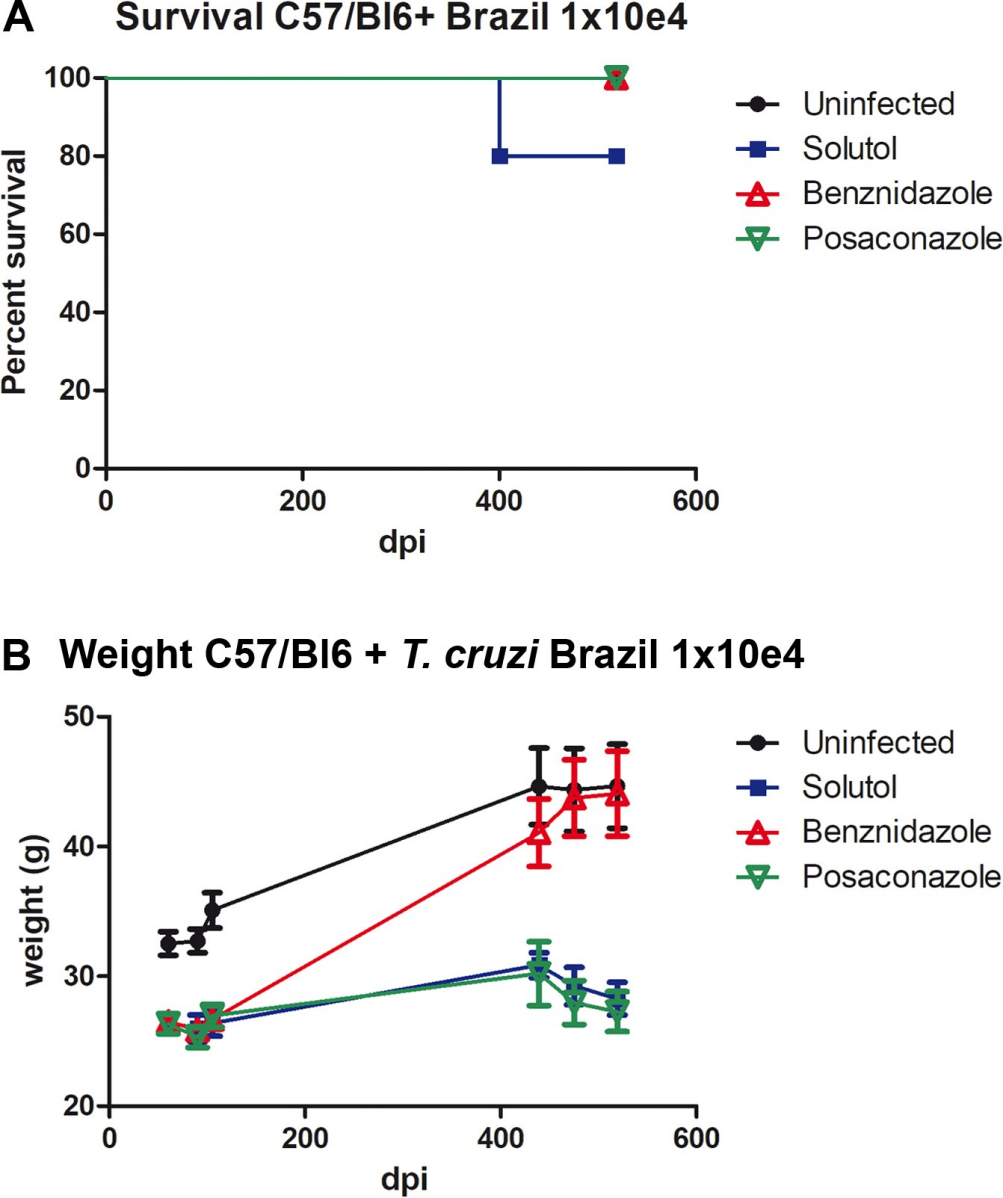
Survival (A) and weight (B) curves from mice infected with *T*. *cruzi*, Brazil strain. Note in Fig 4B that only the benznidazole-treated mice gained significant weight over the one-year period of observation.

**Fig 5:**
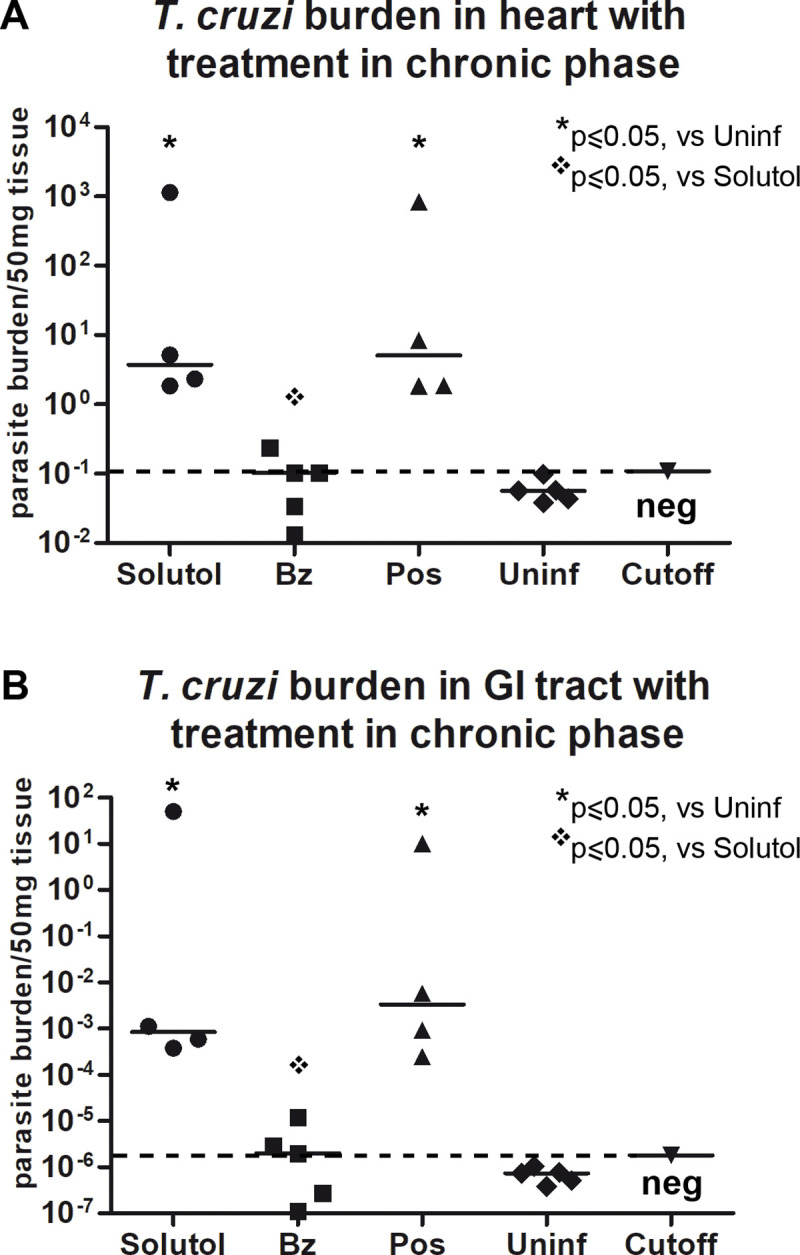
Parasite burden in heart (A) and G.I. tract (B) in chronic model of infection. Only the benznidazole-treated mice were PCR negative at one year in the chronic disease model of *T*.*cruzi* infection. * Statistically significant versus Uninfected samples, p≤ 0.05; ❖ Statistically significant versus Solutol treated mice (vehicle), p≤ 0.05.

**Fig 6:**
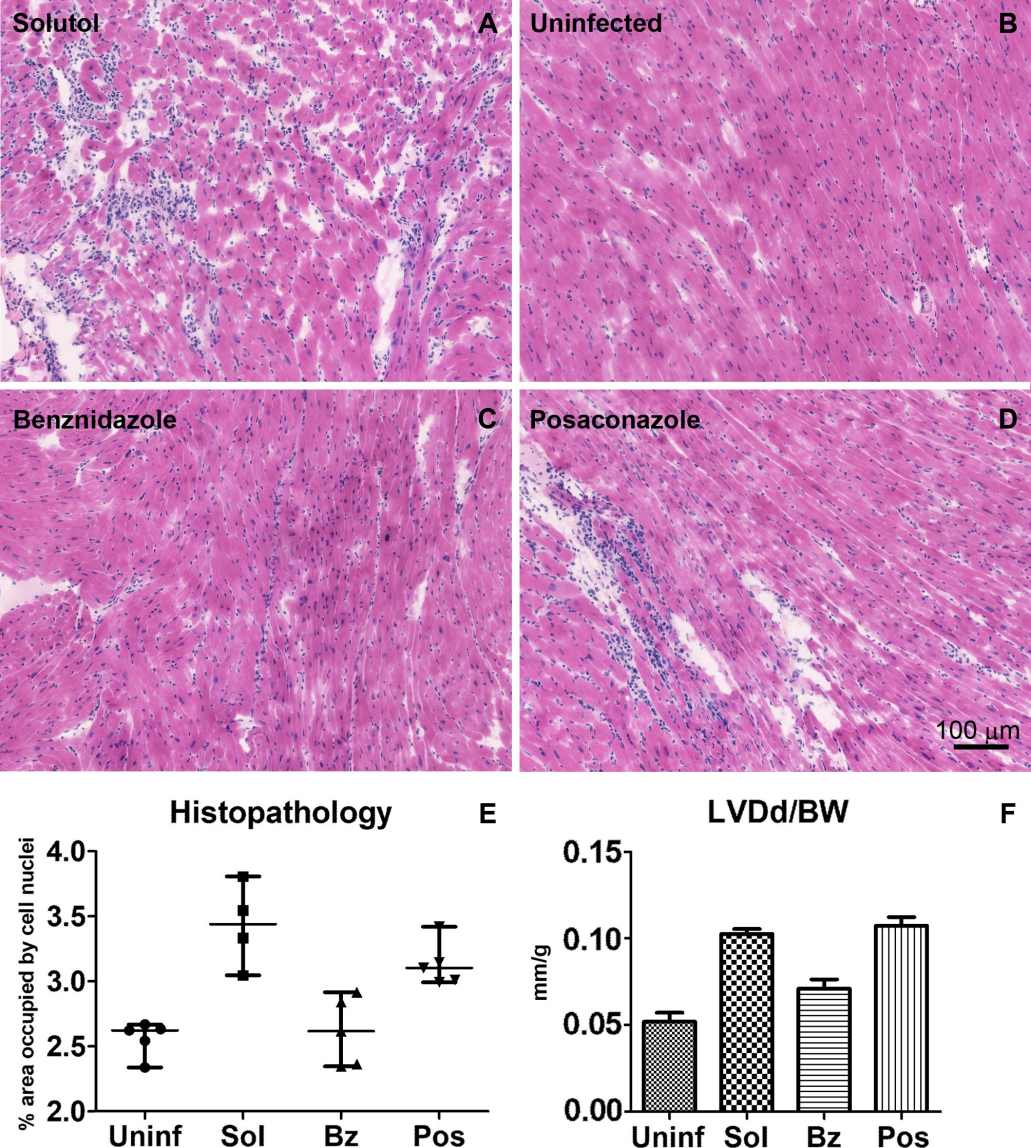
Histopathology analysis of heart tissue of mice infected with *T*. *cruzi*, Brazil strain and treated with compounds in the chronic phase of the disease. (A-E) Examples of inflammatory infiltrates in vehicle-alone treated and posaconazole-treated mice. (F) Echocardiographic analysis of left ventricular size adjusted to mice weight (in mm/g) prior to necropsy of mice in the chronic disease model. Note significant wall hypertrophy in the vehicle-alone and posaconazole-treated mice.

To establish a mouse model of chronic Chagas disease, a different parasite strain (Brazil) and a lower parasite inoculum (10^4^ trypomastigotes) were used. Treatment started 60 days post infection. Survival was not a distinguishing variable as noted in Fig 4A. Therefore, echocardiographic analysis of ventricular size was carried out just before euthanasia at 378 days post treatment. In contrast to the acute infection model, only benznidazole produced a significant increase in the weight of infected mice (Fig 4B). As was the case in the acute model of Chagas Disease, no significant change in activity, food consumption, or gross appearance was noted at one year. However, PCR analysis identified a significant parasite burden in both the heart and G.I. tract for the vehicle alone and posaconazole- treated groups (Fig 5A and 5B). No significant parasite burden, as detected by PCR, was seen in the benznidazole-treated group. Histopathologic analysis of heart tissue confirmed significant inflammation associated with infection in the untreated group, mild inflammation in posaconazole treated mice and no inflammatory infiltrates in the benznidazole- treated mice (Fig 6E). The consequence of significant cardiac infection and inflammation in the vehicle-alone treated, and posaconazole-treated mice was reflected in an increase in ventricular size by echocardiography (Fig 6F).

## Discussion

Evaluation of the efficacy of drug therapy in chronic Chagas disease has been limited by two major issues. The first is the current lack of a validated biomarker to predict clinical progression and response to therapy. The second is the fact that only 30% of patients in the indeterminate chronic phase of Chagas disease will go on to serious and even life-threatening cardiac failure and/or gastrointestinal problems. Not only is it currently difficult or impossible to predict which patients are at risk, but progression can be slow with clinical manifestations becoming apparent only after years or even decades.

While using a mouse model of infection, with the understanding that human disease may have unpredictable differences, one can nevertheless follow disease progression and response to therapy in a reasonable period of time. We therefore evaluated the clinical course of *T*. *cruzi* infection for one year following treatment. Because the lifespan of C57/BL mice has been estimated as 2 to 3 years under laboratory conditions [29], we are likely evaluating the equivalent of decades of infection in humans.

We would like to draw attention to three key results from this study. We understand that these results may only be relevant to the strains of parasites used. First when treatment occurred during the acute phase of infection (six days post infection), all mice survived for one year, gained weight, and showed no gross behavioral or other clinical signs of heart failure. All infected but untreated mice died by 25 days. Interestingly, using this parasite strain and this inbred strain of mice, posaconazole- treatment was more effective than benzidazole-treatment in reducing the severity of cardiac disease. The success of posaconazole treatment in the acute phase of Chagas Disease reported here is also well described in the literature in several mouse models of infection [11,30,31]. Posaconazole treatment was also superior to benznidazole treatment in Swiss mice infected with Y strain of *T*. *cruzi* during acute phase. All of the posaconazole-treated animals tested negative for *T*. *cruzi* through hemoculture, whilst benznidazole only resulted in cure in 50% of the animals[10]. In that study, mice treated with posaconazole also showed less cardiac damage and spleen enlargement than benznidazole treated animals[10]. In addition, in one clinical case of a patient with systemic lupus with reactivation of chronic Chagas infection by immunossupressive treatment, posaconazole was superior to benznidazole[12]. Benznidazole produced a reduction, but not an elimination of *T*. *cruzi*, whereas treatment with posaconazole resulted in consistent negative blood PCR for the parasite throughout 13 months of followup [12]. Results similar to ours were reported in dog infection models. Benznidazole reduced the parasite load in the acute phase, but did not produce sterile cure, and the benznidazole treated animals displayed inflammation and fibrosis in the heart [32]. However, there remains a consensus that benznidazole can be effective in the acute phase of human disease. A review of 56 clinical studies concluded that 82.6% of children and 86.1% acutely infected adult patients were cured of Chagas disease when PCR was used as diagnostic method. The authors did note a high variability in the efficacy of benznidazole-based chemotherapy, depending on the parasite genetic background, and the stage when treatment was performed [33]. Of particular note in our study was the one posaconzole-treated mouse with positive PCR for parasite DNA but no evidence of cardiac infection. While a single data point, this result suggests that there may be instances where sterile cure is not required to prevent progression to cardiac failure. Future clinical trials should therefore evaluate both parameters, taking in consideration general clinical outcomes and not only achievement of sterile cure.

In contrast, benznidazole-treated but not posaconazole-treated mice showed no signs of disease progression when treatment occurred 60 days following infection. While all treated mice survived one year, only the benznidazole-treated mice gained weight and had no evidence of left ventricular hypertrophy by echocardiogram. The stage-specific discrepancy in the performance of posaconazole treatment might be related to the fact that this compound is effective only versus the actively proliferating intracellular form of the parasite [19,34]. In support of our results, other investigators have described that treatment of *T*. *cruzi* infected mice with benznidazole in the chronic phase of infection. While not completely eliminating the parasites, benznidazole treatment resulted in decreased tissue parasitism, reduced myocarditis and lessened electrocardiographical alterations [35]. Studies comparing benznidazole and posaconazole performance using bioluminescent strains of *T*. *cruzi* showed that only benznidazole induced sterile cure in treatment schemes both during acute and chronic phase of *T*. *cruzi* infection [36]. Interestingly, posaconazole was able to significantly reduce parasitemia detection until immunossupresion revealed cryptic parasite nests. After treatment during chronic phase, both compounds significantly prevented splenomegaly, but no other clinical outcome was analyzed [36].

This study was purposely focused on the heart and GI tract because these are the sites of important clinical manifestations of Chagas disease. Also, although the parasite can potentially be found in a variety of tissues, it is also well acknowledged that the GI tract and heart are major sites of persistence of *T*. *cruzi*, specially since both strains used in the assays belong to TcI which showed this preferential localization in the same mouse background [18,37].

The results of these studies have important implications for the design of future clinical trials utilizing benznidazole or posaconazole. Only benznidazole showed any efficacy in reducing cardiac or gastrointestinal manifestations of Chagas Disease in a model of chronic infection. On the other hand, both drugs showed efficacy if treatment began early in infection. This latter result suggests that posaconazole may be a safer alternative to benznidazole in acute Chagas Disease, or more importantly as prophylaxis or treatment of reactivation of infection in immunosuppressed patients such as those undergoing cardiac transplant.
